# Neuropathology of Zika Virus Infection

**DOI:** 10.4172/2314-7326.1000220

**Published:** 2016-06-23

**Authors:** Isaac H Solomon, Danny A Milner, Rebecca D Folkerth

**Affiliations:** Department of Pathology, Brigham and Women’s Hospital, Boston, USA

**Keywords:** Zika virus, Microcephaly, Guillain-Barré syndrome, Neuropathology, Histology

## Abstract

Zika virus (ZIKV) is a member of the Flaviviridae family that had been associated only with mild disease prior to the 2015 outbreak in Brazil. A dramatic increase in reported cases of microcephaly and Guillain-Barré syndrome during this time prompted significant research into possible associations with ZIKV and its neurotropic properties. Infection of neural progenitor cells and organoids have been shown to induce apoptosis and dysregulation of growth, and mouse studies have demonstrated viral replication in brain tissue in adults, as well as vertical transmission resulting in embryonic brain abnormalities. Large case series of clinical and radiological findings of congenital ZIKV infection have begun to be published; however, pathology reports have been limited to two case reports and two small case series. Thus far, the findings have largely been restricted to the brain and include diffuse grey and white matter involvement consisting of dystrophic calcifications, gliosis, microglial nodules, neuronophagia, and scattered lymphocytes. Mild chronic villitis was observed in the placental tissue in some cases, and the remaining organs were essentially uninvolved. Larger, systematic studies, including correlation of histological findings with gestational age at the time of maternal infection, will be required to determine the full range of Zika virus-induced abnormalities and to help guide future clinical decision making.

## Introduction

ZIKV (ZIKV) is a member of the *Flaviviridae* family, first identified in the Zika Forest of Uganda in 1947 [1]. Only rare cases were reported in humans prior to outbreaks on the Yap Island in the Federated State of Micronesia in 2007 and French Polynesia in 2013–2014. Cases were first recognized in Brazil in early 2015, and phylogenetic analysis determined that the virus isolates were most closely related to strains from South East Asia and the Pacific islands, suggesting spread via travelers from affected areas. Until the 2015 outbreak in Brazil, infection with ZIKV was predominantly observed to be asymptomatic or associated with a mild disease course including acute onset of fever with maculopapular rash, arthralgia, myalgia, headache, and conjunctivitis, affecting all age groups. Transmission is typically mediated by *Aedes* species mosquitoes, but virus can also be transmitted perinatally, in utero, sexually, or through transfusion products; virus has been detected in urine, saliva, and breast milk [2–5]. Diagnosis relies on a high degree of suspicion due to overlap in symptoms and geographic region with other arboviral infections, including Chikungunya and Dengue. The diagnosis is confirmed by serology and detection of viral nucleic acids by RT-PCR [6]. Treatment of symptomatic infections is supportive only, as no vaccines are currently available. While deaths from acute infection are rare, ZIKV has been associated with Guillain-Barré syndrome (GBS), which can lead to paralysis and death [7].

## Neurological Disease Associated with ZIKV Infection

The investigation of a 20-fold increase in cases of microcephaly (defined as a head circumference less than 2 standard deviations below the mean), in Brazil in 2015 suggested a possible connection to ZIKV infection during pregnancy, prompting intense research worldwide [8]. Active ZIKV infection has since been reported in over 48 countries and territories in South and North America, and numerous other countries have reported cases in travelers returning from areas with active infection. The Centers for Disease Control and Prevention (CDC) recently released a statement declaring ZIKV to be the proven cause of microcephaly based on the accumulated epidemiological evidence [9]. Through May 19, 2016, 1,384 confirmed and 3,332 suspected ZIKV-associated microcephaly cases and 88,545 suspected and 31,616 confirmed ZIKV infections have been reported in Brazil, suggesting a rate of microcephaly to total cases of 1%–4% [10]. A study of symptomatic pregnant women with laboratory confirmed ZIKV infections reported ultrasound detection of fetal abnormalities in 12/42 (29%) cases [11]. Although microcephaly had not been associated previously with ZIKV infection, retrospective studies of the 2013–2014 outbreak in French Polynesia identified an increase in incidence of microcephaly and other congenital brain abnormalities (8 cases of microcephaly with 8,750 suspected and 383 laboratory confirmed ZIKV infections) during the period of time with active infection [12,13]. The vast majority of cases of congenital ZIKV infections have been reported in Brazil (1384/1401, 98%), while a few scattered cases have been identified in Colombia, Martinique, Panama, Puerto Rico, and the United States [10]. Studies to identify environmental and genetic co-factors impacting the rate and severity of congenital infections are ongoing.

Prior to the current epidemic, neurological illness associated with ZIKV had previously been limited to scattered cases of GBS [7]. In addition to congenital microcephaly and ocular disease in fetuses and infants and a large number of GBS cases in adults, scattered reports of acute myelitis and meningoencephalitis have been published, indicating a broader range of neurological presentations or sequelae may exist [14–16]. The potential neurotropic properties of ZIKV have been known for decades due to early mouse studies [17]. More recent experiments utilizing mice deficient in interferon response, due to knockout of IFN-α/β+/− IFN-γ receptors or treatment with IFNAR1-blocking monoclonal antibodies, have demonstrated rapid viremic dissemination and severe brain pathology [18–21]. Mouse models have also demonstrated vertical transmission resulting in fetal demise with significant brain abnormalities [22,23]. Neurotropism has additionally been confirmed by *in vitro* and *in vivo* experiments in which neural stem cells, brain organoids, and embryonic mouse brains were infected with ZIKV resulting in apoptosis and dysregulation of growth [24–27]. Infection of mice at embryonic day 13.5 resulted in brains smaller than controls, characterized by enlarged lateral ventricles and thinner cortical plates and ventricular and subventricular zones, providing strong evidence that ZIKV can cause microcephaly [27].

## Histopathology of Congenital ZIKV Infection

While larger case series comprising clinical, laboratory, and ultrasound imaging have begun to be published, histopathology of ZIKV-associated microcephaly has been limited to a pair of case reports in the New England Journal of Medicine [28,29], preliminary findings published by the CDC in the Morbidity and Mortality Weekly Report [30], and a small case series from Brazil [31].

Mlakar et al. reported findings from a fetal autopsy from an elective termination performed at 32 weeks gestational age from a mother infected during the 13th week of pregnancy [28]. Ultrasound examinations at 29 and 32 weeks demonstrated intrauterine growth retardation, microcephaly, ventriculomegaly, decreased cerebellar diameter, and calcifications. Gross examination showed microcephaly (small head circumference), microencephaly (small brain), a small cerebellum and brain stem, near-complete agyria, and calcifications in the cortex and subcortical white matter. In addition to dystrophic calcifications, microscopic analysis revealed diffuse gliosis, activated microglial cells and macrophages in the cerebral white and gray matter, scattered lymphocytes in the subcortical white matter, and normal cerebellum, brain stem, and spinal cord (with the exception of Wallerian degeneration of descending spinal tracts). The placenta had focal calcification in the villi without inflammation, and the remaining organs were unaffected. ZIKV in brain tissue was demonstrated by RT-PCR and electron microscopy.

Driggers et al. reported fetal autopsy findings from an elective termination performed at 21 weeks gestational age from a mother infected during the 11th week of pregnancy [29]. Decrease in fetal head circumference percentile was observed by serial ultrasounds, as well as enlarged ventricles, a thinned cerebral mantle, absence of cavum septi pellucidi, but no calcifications. Gross and microscopic examinations of the fetus were limited due to severe autolysis, but showed apoptosis of intermediately differentiated post-migratory neurons in the neocortex with early mineralization, severe volume loss and extensive axonal rarefaction of subventricular zone and white matter, and scattered microglial aggregates. No abnormalities were detected in the eyes or spinal cord. ZIKV in brain tissue was confirmed by electron microscopy of brain tissue and by RT-PCR of brain, placenta, amniotic fluid, and maternal serum, and by virus isolation in SK-N-SH (human neuroblastoma) and Vero E6 (monkey kidney epithelial) cell lines.

Martines et al. described preliminary findings from two miscarriages at 11 and 13 weeks gestation and two newborns with microcephaly that were born at 36 and 38 weeks gestational age and died within 20 hours of birth [30]. ZIKV was detected in all four cases by RT-PCR. Immunohistochemistry using a polyclonal anti-ZIKV antibody identified virus in glial cells and neurons in the brain of one of the newborns and in the chorionic villi in one of the miscarriages. Microscopic findings in the newborns included calcifications in brain parenchyma with microglial nodules, gliosis, and cell degeneration and necrosis, with normal placenta and other organs. Placental tissue from one of the miscarriages showed patchy intervillositis and villitis, with calcifications, fibrosis, and fibrin deposition.

Noronha et al. described findings from five cases in Brazil including one fetus, three deceased infants with microcephaly, and the placenta from one living infant without microcephaly [31]. The first case consisted of a fetal demise at 12 weeks gestational age following maternal infection during the seventh week of pregnancy. No fetal tissues were identified. Examination of placental tissue revealed histiocyte-predominant chronic villitis and edema. Anti-flavivirus antibody 4G2 (monoclonal antibody raised against Dengue virus with broad flavivirus cross-reactivity) showed positive cytoplasmic staining limited to histiocytes, suggesting limited or no replication in trophoblastic epithelium.

RT-PCR confirmed the presence of ZIKV. Three cases of infants with microcephaly were delivered between 35 and 40 weeks gestational age and died within one day of birth following maternal infection one month prior to pregnancy, during the third month of pregnancy, and at an unknown time. Findings were similar in the three cases, predominated by brain tissue with perivascular mononuclear inflammatory cells, microglial nodules, neuronophagia, gliosis, and microcalcifications. Placental tissue showed distal villous hypoplasia, increased macrophages, plasma cells, and lymphocytes, and focal calcifications. Liver showed increased extramedullary hematopoiesis, and liver, lung, kidney, spleen, heart, and adrenal gland showed vascular congestion. IHC with 4G2 antibody demonstrated positive cytoplasmic staining in some glial cells, and RT-PCR for ZIKV was positive in placenta and brain. The final case consisted of an infant born at nine months without microcephaly to a mother infected during the eighth month of pregnancy. No histologic examination was reported, but ZIKV RNA was detected in placental tissue.

The initial ZIKV histopathological reports allow for limited generalizations, but thus far it appears that the major findings are restricted to the brain with variable placental involvement, and that infections earlier in pregnancy are associated with more severe pathology. The virus has been shown to infect keratinocytes and dendritic cells at the site of a mosquito bite and to then spread via blood stream and lymphatics [32]; however, the mechanism by which fetal infection occurs and microcephaly develops is unknown. Despite the limited histologic findings, the placenta likely plays a role in pathogenesis, which may include direct conveyance of ZIKV to the fetus or the mounting of a response leading to brain abnormalities, or both [33].

## Conclusions

Understanding the full spectrum of ZIKV-associated congenital abnormalities will require the systematic examination of fetal and neonatal autopsy tissues and placentas from living infants with microcephaly at many gestational ages at maternal infection. This information will aid both in understanding the mechanism of ZIKV pathogenesis and determine the earliest detectable and non-reversible findings in order to better guide clinical decision-making. To this end, we recommend extensive sampling of all organs (particularly brain and placenta) of miscarriages in mothers with potential ZIKV exposure, and all fetuses or deceased infants with microcephaly or with mothers with potential ZIKV exposure. Examination of the brain tissue should be undertaken by a neuropathologist with expertise in perinatal brain pathology, and representative tissue, including serum from the mother, if not already known to be ZIKV positive, should be sent to reference centers such as the CDC (via applicable state reference laboratory procedures) for confirmation and further study.
